# Sodium-Glucose Co-transporter 2 Inhibitors in the Failing Heart: a Growing Potential

**DOI:** 10.1007/s10557-020-06973-3

**Published:** 2020-04-30

**Authors:** Dulce Brito, Paulo Bettencourt, Davide Carvalho, Jorge Ferreira, Ricardo Fontes-Carvalho, Fátima Franco, Brenda Moura, José Carlos Silva-Cardoso, Rachel Tavares de Melo, Cândida Fonseca

**Affiliations:** 1 Department of Cardiology, Centro Hospitalar Universitário Lisboa Norte, Av. Prof. Egas Moniz, 1649-035 Lisboa, Portugal; 2 CCUL, Faculdade de Medicina, Universidade de Lisboa, Av. Prof. Egas Moniz, 1649-035 Lisboa, Portugal; 3 Department of Internal Medicine, Hospital CUF Porto, Porto, Portugal; 4 Faculdade de Medicina, Universidade do Porto, Porto, Portugal; 5 Department of Endocrinology, Diabetes and Metabolism, Centro Hospitalar, Universitário de São João, Porto, Portugal; 6 Instituto de Investigação e Inovação em Saúde, Universidade do Porto, Porto, Portugal; 7 Department of Cardiology, Hospital de Santa Cruz, Centro Hospitalar de Lisboa Ocidental, Lisbon, Portugal; 8 Department of Cardiology, Centro Hospitalar Vila Nova de Gaia/Espinho, Espinho, Portugal; 9 Department of Surgery and Physiology, Cardiovascular Investigation Unit, Faculdade de Medicina, Universidade do Porto, Porto, Portugal; 10 Department of Cardiology, Centro Hospitalar e Universitário de Coimbra, Coimbra, Portugal; 11 Department of Cardiology, Hospital das Forças Armadas-Pólo do Porto, Porto, Portugal; 12 CINTESIS-Cardiocare, Center for Health Technology and Services Research, Porto, Portugal; 13 Department of Cardiology, Centro Hospitalar Universitário de São João, Porto, Portugal; 14 Medical Department, Boehringer Ingelheim, Lisboa, Portugal; 15 Heart Failure Clinic, Hospital São Francisco Xavier, Centro Hospitalar de Lisboa Ocidental, Lisboa, Portugal; 16 NOVA Medical School, Faculdade de Ciências Médicas, Universidade Nova de Lisboa, Lisboa, Portugal

**Keywords:** SGLT2i, Diabetes, Heart failure, Cardiovascular risk, Cardiovascular outcomes trials

## Abstract

Sodium-glucose co-transporter 2 inhibitors (SGLT2i) are a new drug class designed to treat patients with type 2 diabetes (T2D). However, cardiovascular outcome trials showed that SGLT2i also offer protection against heart failure (HF)–related events and cardiovascular mortality. These benefits appear to be independent of glycaemic control and have recently been demonstrated in the HF population with reduced ejection fraction (HFrEF), with or without T2D. This comprehensive, evidence-based review focuses on the published studies concerning HF outcomes with SGLT2i, discussing issues that may underlie the different results, along with the impact of these new drugs in clinical practice. The potential translational mechanisms behind SGLT2i cardio-renal benefits and the information that ongoing studies may add to the already existing body of evidence are also reviewed. Finally, we focus on practical management issues regarding SGLT2i use in association with other T2D and HFrEF common pharmacological therapies. Safety considerations are also highlighted. Considering the paradigm shift in T2D management, from a focus on glycaemic control to a broader approach on cardiovascular protection and event reduction, including the potential for wide SGLT2i implementation in HF patients, with or without T2D, we are facing a promising time for major changes in the global management of cardiovascular disease.

**Electronic supplementary material:**

The online version of this article (10.1007/s10557-020-06973-3) contains supplementary material, which is available to authorized users.

## Diabetes and Heart Failure

Type 2 diabetes (T2D) and heart failure (HF) are common and often coexisting conditions, with a harmful relationship. HF affects at least 26 million people worldwide, but projections regarding rising prevalence for the next decades are alarming, namely due to an ageing population and to the expected increase in HF with preserved ejection fraction (HFpEF) [1–5]. Currently, despite advances in HF treatment, mortality can reach 50% at five years, exceeding that observed in most common malignant neoplasms [6, 7]. Hospitalizations contribute to the high morbidity in HF and account for most of its costs, which are likely to rise dramatically [3, 4, 8].

The prevalence of T2D has nearly doubled since 1980 and is expected to affect nearly 580 million individuals worldwide in 10 years, and 700 million by 2045 [9, 10]. T2D is a major risk factor for the development of cardiovascular disease (CVD), its main cause of morbidity and mortality [11, 12].

The relationship between T2D and HF has been well established since the Framingham study, which reported a 2- and 5-fold higher risk of HF in men and women with T2D, respectively, compared with individuals without T2D [13]. More recently, the Reykjavik study described a 12% prevalence of HF in the T2D population vs. 3% in individuals without T2D [14].

T2D is associated with cardiac structural changes including increased interstitial fibrosis, left ventricular hypertrophy, endothelial dysfunction, microangiopathic processes and inflammation, factors that confer a higher risk for developing HF with or without a reduced ejection fraction (rEF) [15]. T2D adversely affects outcomes amongst patients with HF, has a substantial influence on the costs of managing HF patients, extends hospital stay and worsens prognosis [4, 16]. On the other hand, HF increases the risk of fatal and non-fatal cardiovascular (CV) events in T2D patients [17].

Sodium-glucose co-transporter 2 inhibitors (SGLT2i) have emerged as a new class of drugs designed to treat patients with T2D, but have also been shown to be protective against HF-related events and CV mortality. Herein, we present a comprehensive, evidence-based overview concerning the use of SGLT2i in patients with or without T2D at risk for CV events, focusing on HF outcomes. Additionally, we perform a critical analysis of the SGLT2i cardiovascular outcome trials (CVOTs) and discuss the SGLT2i possible translational mechanisms behind the clinical outcomes, with an overview of the ongoing SGLT2i functional capacity, mechanistic and HF outcomes trials. Finally, we present a summary of practical considerations regarding the co-administration of SGLT2i and common therapies used in T2D and HFrEF, as well as management of safety issues, based on expert opinion and current recommendations.

## Improving Prognosis: the Clinical Research Arena

### SGLT2i Cardiovascular Outcome Trials in T2D Patients

Due to concerns for possible adverse CV outcomes with antidiabetic agents, both the Food and Drug Administration (FDA) and the European Medicines Agency require that all new glucose-lowering drugs demonstrate CV safety in T2D patients. This is now tested in CVOTs that analyse drug safety in terms of MACE (major adverse cardiovascular events), which include CV death, non-fatal myocardial infarction (MI) and non-fatal stroke (3-point MACE). Surprisingly, HF outcomes, which can be precipitated by some antidiabetic drugs [18], are not included as a mandatory component of composite endpoints [19].

A paradigm shift in T2D management emerged when CVOTs with SGLT2i [20–22] demonstrated that these drugs are not only safe in terms of 3-point MACE but may also be beneficial in HF-related and renal events, regardless of the presence of atherosclerotic CVD (ASCVD) or HF at baseline.

#### The EMPA-REG OUTCOME Trial

The EMPA-REG OUTCOME trial analysed the outcomes of the SGLT2i empagliflozin vs. placebo in patients with T2D and established CVD at baseline, and demonstrated the superiority of empagliflozin in reducing the risk of 3-point MACE, with significant reductions in CV death, all-cause death and in hospitalization for HF (HHF). Observed benefits were related to a decrease in incident HF events rather than to prevention of ischemic CV events [20]. These unexpected results led empagliflozin to become the first glucose-lowering drug approved for CV death protection in T2D patients.

Post hoc analyses of the EMPA-REG OUTCOME trial revealed that besides the higher incident rates of HHF, CV death and all-cause mortality in patients with HF at baseline compared with patients without HF, the risk reductions of these outcomes with empagliflozin were consistent in both subgroups (hazard ratio (HR): 0.67, 95% confidence interval (CI) 0.47–0.97 in patients with HF burden (defined as HF at baseline, HHF or incident HF without hospitalization during the trial); HR 0.63, 95% CI 0.48–0.84 in patients without HF burden) [23]. The observed benefit with empagliflozin extends to the two causes of cardiac death in HF: sudden death and pump failure [24].

#### The CANVAS Program and the DECLARE-TIMI 58 Trial

The CANVAS program [21], which included the CANVAS and CANVAS-R (renal) studies, assessed the CV safety and efficacy of canagliflozin in patients with T2DM and established CVD or at least two risk factors for CVD. The DECLARE-TIMI 58 trial [25] compared dapagliflozin vs. placebo in patients with T2DM with either established ASCVD or multiple risk factors for ASCVD. These trials showed that both SGLT2i also reduced the composite endpoint of CV death and HHF in T2D patients, with similar results observed with dapagliflozin for patients with and without HF at baseline [22]. The results for both trials were driven by a reduction in HHF, with no significant reduction in CV death alone.

Table 1 presents a summary of the CVOTs with SGLT2i. These CVOTs have different designs and inclusion criteria, and therefore are difficult to compare; additionally, the definition of CV risk is variable across studies, and there is no information regarding baseline LV ejection fraction (LVEF).

**Table 1:** Summary of cardiovascular outcome trials with SGLT2i in patients with type 2 diabetes

	EMPA-REG outcome [20] Empagliflozin vs. placebo	CANVAS [21] Canagliflozin vs. placebo	DECLARE-TIMI 58 [22] Dapagliflozin vs. placebo	VERTIS CV[26] Ertugliflozin vs. placebo
Study design and sample main features	7028 patients with T2D, 99.4% with established CVD 57% > 10 yr T2D and 25.1% 5–10 yr T2D Median follow-up 3.1 yr	9734 patients with T2D, 65.6% with established CVD Mean T2D duration 13.5 yr Median follow-up 2.4 yr	17,160 patients with T2D, 40.5% with established CVD Median T2D duration 11 yr Median follow-up of 4.2 yr	8246 patients with T2D, 100% with established ASCVD Mean T2D duration 12.9 years
Primary endpoint: 3P-MACE (CV death, MI or stroke)	37.4 vs. 43.9 per 1000 pt-yrs HR 0.86 (95% CI, 0.74–0.99; *p* < 0.001 for noninferiority and *p* = 0.04 for superiority)	26.9 vs. 31.5 per 1000 pt-yrs HR 0.86 (95% CI, 0.75–0.97; *p* < 0.001 for noninferiority and *p* = 0.02 for superiority)	22.6 vs. 24.2 per 1000 pt-yrs HR 0.93 (95% CI, 0.84–1.03; *p* = 0.17 for superiority)	Estimated completion December 2019
CV death or HHF	19.7 vs. 30.1 per 1000 pt-yrs HR 0.66 (95% CI, 0.55–0.79; *p* < 0.001)	16.3 vs.20.8 per 1000 pt-yrs HR 0.78 (95% CI, 0.67–0.91; *p* = NA)	12.2 vs. 14.7 per 1000 pt-yrs HR 0.83 (95% CI, 0.73–0.95; *p* = 0.005)	
CV death	12.4 vs. 20.2 per 1000 pt-yrs HR 0.62 (95% CI, 0.49–0.77; *p* < 0.001)	11.6 vs. 12.8 per 1000 pt-yrs HR 0.87 (95% CI, 0.72–1.06; *p* = ns)	7.0 vs.7.1 per 1000 pt-yrs HR 0.98 (95% CI, 0.82–1.17; *p* = ns)	
HHF	9.4 vs. 14.5 per 1000 pt-yrs HR 0.65 (95% CI, 0.50–0.85; *p* = 0.002)	5.5 vs. 8.7 per 1000 pt-yrs HR 0.67 (95% CI, 0.52–0.87; *p* = NA)	6.2 vs. 8.5 per 1000 pt-yrs HR 0.73 (95% CI, 0.61–0.88; *p* = NA)	
Death by any cause	19.4 vs. 28.6 per 1000 pt-yrs HR 0.68 (95% CI, 0.57–0.82; *p* < 0.001)	17.3 vs. 19.5 per 1000 pt-yrs HR 0.87 (95% CI, 0.74–1.01; *p* = ns)	15.1 vs. 16.4 per 1000 pt-yrs HR 0.93 (95% CI, 0.82–1.04; *p* = ns)	
Safety data	Higher incidence of genital infection (6.4% vs. 1.8%; < 0.001)	Higher incidence of amputation (6.3 vs. 3.4 per 1000 pt-yrs; HR 1.97; 95% CI, 1.41–2.75) Higher incidence bone fracture (15.4 vs. 11.9 per 1000 pt-yrs; HR 1.26; 95% CI, 1.04–1.52)	Higher incidence of diabetic ketoacidosis (0.3% vs. 0.1%; HR 2.18; 95% CI, 1.10–4.30; *p* = 0.02) Higher incidence genital infection (0.9% vs. 0.1%; HR 8.36; 95% CI, 4.19–16.68; *p* < 0.001)	

*Abbreviations*: *3P-MACE*, 3-point major adverse cardiovascular events; *ASCVD*, atherosclerotic cardiovascular disease;*CI*, confidence interval; *CV*, cardiovascular; *CVD*, cardiovascular disease; *HHF*, hospitalization for heart failure; *HR*, hazard ratio; *MI*, myocardial infarction; *NA*, not available; *ns*, not significant; *pt-yrs*, patient-years; *T2D*, type 2 diabetes mellitus; *yr*, years

#### Meta-analyses

A question that remains unresolved is whether the effects are consistent across the SGLT2i class, or whether pharmacologic differences between the drugs may translate into differences in clinical efficacy and safety outcomes.

Two meta-analyses on SGLT2i CVOTs have been recently published, aiming to better estimate the class effect of these drugs on CV outcomes [27, 28]. The most recent meta-analysis showed a consistent class effect of SGLT2i in reducing HHF in patients with or without baseline CVD, as well as a consistent effect on preventing the progression of renal disease [27].

Overall, SGLT2i reduced the risk of MI by 11% and the risk of CV death by 16%, although significant heterogeneity in CV death was observed between trials. Similarly, all-cause mortality was reduced by 15%, again with significant heterogeneity. When only patients with ASCVD were compared within trials (excluding patients with multiple CV risk factors in the CANVAS and DECLARE-TIMI 58 trials), empagliflozin was the only that showed significant reductions on CV death and all-cause mortality. Similarly, an increased risk in amputations and fractures was only noted with canagliflozin [27].

It is possible that either pharmacologic differences within the class, or differences in the baseline risk within the study populations, may be responsible for the observed heterogeneity in mortality outcomes. The consistently higher event rates in the placebo group in EMPA-REG (compared with the placebo arms in CANVAS and DECLARE-TIMI 58) reflect a higher risk population in the former (also when comparing the ASCVD groups only), which might account for the differences observed between trials.

The estimated glomerular filtration rate (eGFR) cut-off for EMPA-REG was less restrictive, thus allowing for patients with more severe renal dysfunction to be included in the trial. The percentage of patients with eGFR < 60 mL/min was 25.9% in EMPA-REG compared with 20.1% in CANVAS and 7.4% in DECLARE-TIMI 58. Nonetheless, in a subanalysis conducted to determine the impact of eGFR on CV death in EMPA-REG, a consistent effect on CV mortality was observed, independent of baseline eGFR [20, 29, 30].

Furthermore, the recent CREDENCE trial tested canagliflozin vs. placebo in 4200 T2D patients with nephropathy, an eGFR of 30 to < 90 mL/min and albuminuria [31]. Over 50% of the population had established CVD, and 14.8% had HF at baseline. During a median follow-up of 2.62 years, canagliflozin did not significantly reduce CV death alone, despite a nominally non-significant *p* value (HR 0.78, 95% CI 0.61–1.00, *p* = 0.05), or all-cause death ((HR 0.83, 95% CI 0.68–1.02, *p* = not available (NA)), but showed a pronounced reduction in HHF (HR 0.61, 95% CI 0.47–0.80, *p* < 0.001).

In aggregate, these findings suggest that in patients with greater renal dysfunction, SGLT2i confer even higher reductions in HHF, as also suggested by the meta-analysis results [27]. However, the degree of renal dysfunction or presence of established CVD does not appear to fully explain the observed heterogeneity in terms of mortality amongst the three published SGLT2i CVOTs.

Based on this heterogeneity, the 2019 European Society of Cardiology (ESC) Guidelines [32] on diabetes, pre-diabetes and CVD, developed in collaboration with the European Association for the Study of Diabetes (EASD), has given empagliflozin a class IB recommendation to reduce the risk of death in patients with T2D and CVD. In addition, empagliflozin, dapagliflozin and canagliflozin are recommended in patients with T2D and CVD or at very high/high CV risk, to reduce CV events, as first-line antidiabetic therapy in naive patients, not previously treated with metformin [32]. This recommendation is criticized, namely by the convincing beneficial effects (HbA1c 6.5–7.5%) (glycated haemoglobin) of early combination therapy [33].

A CVOT with the SGLT2i ertugliflozin [26] is currently underway, with results expected in the near future (Table 1).

#### SGLT2i Effects on HF Outcomes in T2D Patients

Additional subanalyses of the three abovementioned CVOTs [20–22] have been published, revealing further data concerning SGLT2i effects on HF outcomes in patients with T2D.

An analysis of the CANVAS program showed that canagliflozin reduced the overall risk of HF events in patients with T2D and high CV risk, with no clear difference in effects on HFrEF vs. HFpEF events [34].

A recent analysis of the DECLARE-TIMI 58 trial investigated the efficacy of dapagliflozin in T2D patients considering baseline HF status [25]. In patients with T2D and baseline HFrEF, dapagliflozin reduced HHF, CV death and all-cause mortality, whereas in patients with T2D without baseline HFrEF, the only reduction observed was in HHF [25].

### SGLT2i HF-Dedicated Outcomes Trials in Patients with or without T2D

More recently, the DAPA-HF trial results were published [35]. The trial included 4744 HFrEF patients with our without T2D followed over a median of 18.2 months. It was demonstrated that dapagliflozin 10 mg daily significantly reduced the primary composite endpoint of worsening HF (including HHF or urgent HF visits) and CV death in a population highly treated with background disease-modifying HF therapies (HR 0.74, 95% CI 0.65–0.85, *p* = 0.001), either in patients with (HR 0.75, 95% CI 0.63–0.90, *p* = NA) or without diabetes (HR 0.73, 95% CI 0.60–0.88, *p* = NA) [36]. The number of patients needed to treat (NNT) with dapagliflozin to prevent one primary event during the trial duration was 21 (95% CI 15–38). Importantly, in a post hoc analysis including patients on concomitant sacubitril/valsartan therapy at baseline (nearly 10% of the trial population), the HR for the primary outcome was consistent amongst patients on- or off-sacubitril/valsartan. Despite the low percentage of patients treated with sacubitril/valsartan at baseline, it appears that the benefits of SGLT2i therapy are additive to those afforded by neurohormonal modulating agents. Moreover, possible heterogeneity was observed according to New York Heart Association (NYHA) functional class, showing greater treatment benefit in class II patients, compared with class III or IV [35]. Regarding safety, the occurrence of adverse events (AEs) was low and similar between dapagliflozin and placebo, except for significantly more severe renal adverse events (AEs) in the placebo group (2.7% vs. 1.6%, *p* = 0.009) [36].

Table 2 and Table 3 summarize the ongoing HF-dedicated outcomes [36–38] and functional capacity clinical trials with SGLT2i, which will enhance the body of evidence for these agents in HF populations.

**Table 2:** Summary of published or ongoing dedicated heart failure outcome trials of SGLT2i

	EMPEROR-Preserved [37]	EMPEROR-Reduced	DELIVER	DAPA-HF [36, 38]	Hamad Medical Corporation (ISS)
NCT number	03057951	03057977	03619213	03036124	03794518
Active substance/comparator	Empagliflozin/placebo		Dapagliflozin/placebo	Dapagliflozin/placebo	Pioglitazone + dapagliflozin/placebo
Population	HFpEF	HFrEF	HFpEF with or without T2D	HFrEF with or without T2D	HFpEF with T2D
	With or without T2D				
Sample size	5750	3600	4700	4744	648
Key inclusion criteria	– Chronic HF – Elevated NT-proBNP – eGFR ≥ 20 mL/min/1.73 m^2^ – BP ≥ 100 mmHg		– Symptomatic HFpEF – Elevated NT-proBNP – eGFR ≥ 25 mL/min/1.73 m^2^ – Ambulatory and hospitalized patients – HFpEF (LVEF > 40%)	– Symptomatic HFrEF – Elevated NT-proBNP – eGFR ≥ 30 mL/min/1.73 m^2^ – BP ≥ 95 mmHg – HFrEF (LVEF ≤ 40%)	– T2D – Drug naïve or on stable dose of antidiabetic therapy for 3 months – Hospitalized for HFpEF – eGFR > 60 mL/min – HFpEF (LVEF > 50%)
	HFpEF (LVEF > 40%)	HFrEF (LVEF ≤ 40%)			
Primary endpoint	Time to first event of adjudicated CV death or adjudicated HHF		Time to first occurrence of CV death, HHF or urgent HF visit	Time to first occurrence of CV death, HHF or urgent HF visit	Time to first HHF after starting intervention (3 years)
Key secondary endpoints	– Individual components of primary endpoint – Time to all-cause mortality – All-cause hospitalization – Time to first occurrence of chronic dialysis, kidney transplant or sustained reduction of eGFR – Change from baseline in KCCQ		– Total number of CV death or HHF – Time to death from any cause – Proportion of patients with worsened NYHA class – Change from baseline in KCCQ	– Total number of CV death or HHF – Time to death from any cause – Composite of ≥ 50% sustained eGFR decline, ESRD or kidney death – Change from baseline in KCCQ	– Number of all-cause mortality (total mortality, incidence of acute coronary syndrome and non-fatal CVA)
Results/status	Estimated completion November 2020	Estimated completion July 2020	Estimated completion June 2021	Primary outcome, 16.3% in the dapagliflozin group and 21.2% in the placebo group (HR 0.74; 95% CI 0.65–0.85). Risk of the primary endpoint: HR 0.7 3, 95% CI 0.60–0.88 in patients without T2D and HR 0.75, 95% CI 0.63–0.85 in patients with T2D. Low number of AEs with no differences between groups.	Estimated completion December 2021

*Abbreviations*: *AE*, adverse events; *BP*, blood pressure; *CI*, confidence interval; *CV*, cardiovascular; *CVA*, cerebrovascular accidents; *eGFR*, estimated glomerular filtration rate; *ESRD*, end-stage renal disease; *HF*, heart failure; *HFpEF*, heart failure with preserved ejection fraction; *HFrEF*, heart failure with reduced ejection fraction; *HHF*, hospitalization for heart failure; *HR*, hazard ratio; *KCCQ*, Kansas City Cardiomyopathy Questionnaire; *LVEF*, left ventricular ejection fraction; *NT-proBNP*, N-terminal pro–B-type natriuretic peptide; *NYHA*, New York Heart Association classification; *T2D*, type 2 diabetes mellitus

**Table 3:** Summary of ongoing dedicated heart failure functional capacity trials of SGLT2i

	EMPERIAL-Preserved [39]	EMPERIAL-Reduced [39]	Effects of empagliflozin on exercise capacity and LV diastolic function in patients with HFpEF and T2D	PRESERVED-HF	DEFINE-HF [40]	DETERMINE-Preserved	DETERMINE-Reduced	Treatment of diabetes in patients with systolic HF
NCT number	03448406	03448419	03753087	03030235	02653482	03877224	03877237	02920918
Active substance/comparator	Empagliflozin/Placebo		Empagliflozin/none (unmasked)	Dapagliflozin/placebo	Dapagliflozin/placebo	Dapagliflozin/placebo		Canagliflozin/sitagliptin
Population	HFpEF	HFrEF	HFpEF with T2D	HFpEF with or without T2D	HFrEF with or without T2D	HFpEF	HFrEF	HFrEF with T2D
	With or without T2D					With or without T2D		
Sample size	300	300	100	320	263	400 (estimated)	300 (estimated)	36
Key inclusion criteria	– Chronic HF NYHA class II–IV – Walking distance in the 6MWT ≤ 350 m		– Aged 45–80 years – T2D (HbA1c ≥ 6.5% and ≤ 10%)	– HF NYHA class II–IV – HFpEF (LVEF > 40%) and elevated NT-proBNP	– HF NYHA class II–IV – No change in diuretic management for at least 1 week prior to enrolment – BNP ≥ 100 pg/mL and/or NT-proBNP ≥ 400 pg/mL at enrolment – HFrEF (LVEF ≤ 40%) and elevated NT-proBNP	– Aged ≥ 40 years – HF NYHA class II–IV – Evidence of structural heart disease – 6MWT ≥ 100 m and ≤ 425 m – HFpEF (LVEF > 40%) and elevated NT-proBNP	– HF NYHA class II–IV – 6MWT ≥ 100 m and ≤ 425 m – HFrEF (LVEF ≤ 40%) and elevated NT-proBNP	– T2D and HF NYHA class II–III – RER > 1.00 – HFrEF (LVEF ≤ 40%)
	– HFpEF (LVEF > 40%) and elevated NT-proBNP	– HFrEF (LVEF ≤ 40%) and elevated NT-proBNP						
Aim	To evaluate the effect of empagliflozin 10 mg vs. placebo on exercise ability using the 6MWT in patients with HFrEF or HFpEF		Effects of empagliflozin on exercise capacity and LV diastolic function in patients with HFpEF and T2D	Effects of dapagliflozin on biomarkers, symptoms and functional status in patients with HFpEF	Dapagliflozin effect on symptoms and biomarkers in patients with HF	Effect of dapagliflozin on exercise capacity in patients HFpEF	Effect of dapagliflozin on exercise capacity in patients with HFrEF	Treatment of diabetes in patients with systolic HF
Primary endpoint	Change from baseline to week 12 in exercise capacity (6MWT)		Change in 6MWT at 24 weeks	Change from baseline in NT-proBNP at 6 and 12 weeks	6-week and 12-week NT-proBNP levels Composite of elevation in HF-specific health status by at least 5 points in the KCCQ score or ≥ 20% decrease in NT-proBNP levels	Change from baseline to week 16 in 6MWT		Change from baseline aerobic exercise capacity at 12 weeks measured by cardiopulmonary exercise test
Results/status	Estimated completion October 2019		Estimated completion May 2020	Estimated completion February 2021	No significant difference in average NT-proBNP with dapagliflozin vs. placebo (1133 pg/dL, 95% CI 1036–1238, vs. 1191 pg/dL, 95% CI 1089–1304, *p* = 0.43). Composite of elevation in HF health status and decrease in NT-proBNP levels, the OR effect for dapagliflozin was 1.8 (95% CI, 1.03–3.06, *p* = 0.039).	Estimated completion February 2020	Estimated completion January 2020	Estimated completion September 2018 No results available

*Abbreviations*: *6MWT*, six-minute walking test; *BNP*, B-type natriuretic peptide; *CI*, confidence interval; *HF*, heart failure; *HbA1c*, haemoglobin A1c (glycated haemoglobin); *HFpEF*, heart failure with preserved ejection fraction; *HFrEF*, heart failure with reduced ejection fraction; *KCCQ*, Kansas City Cardiomyopathy Questionnaire; *LV*, left ventricle; *LVEF*, left ventricular ejection fraction; *NT-proBNP*, N-terminal pro–B-type natriuretic peptide; *NYHA*, New York Heart Association classification; *OR*, odds ratio; *RER*, respiratory exchange ratio; *T2D*, type 2 diabetes mellitus

## From Clinical Trials to the Real World

The SGLT2i positive impact on CV outcomes observed in CVOTs, specifically regarding HHF, was also observed in the real-world evidence (RWE) studies CVD-REAL and EMPRISE [41, 42].

CVD-REAL study included 309,056 T2D patients with or without CVD at baseline, newly treated with SGLT2i or other glucose-lowering drugs, from registries within six countries. All primary analyses showed a benefit of SGLT2i over other glucose-lowering drugs: HHF (HR 0.61, 95% CI 0.51–0.73, *p* < 0.001); all-cause mortality (HR 0.49, 95% CI 0.41–0.57, *p* < 0.001), and HHF or death by any cause composite outcome (HR 0.54, 95% CI 0.48–0.60, *p* < 0.001) [42].

The ongoing EMPRISE study aims to assess empagliflozin’s effectiveness, safety, and healthcare utilization in routine care in the USA, including data from 232,000 T2D patients newly initiated on empagliflozin or sitagliptin. After five months follow-up of the nearly 32,000 matched patients, empagliflozin decreased the risk of HHF by 50% (HR 0.50, 95% CI 0.28–0.91, *p* = NA), with consistent results in patients with or without baseline CVD [41].

The available data on SGLT2i, both from CVOTs and from RWE, has undoubtedly shifted the paradigm of T2D management in clinical practice from a focus on glycaemic control to a broader approach on CV event reduction, and also as a potential new class of drugs for HF treatment even in people without T2D. Given the impressive cardioprotective effects of SGLT2i, the results of the DAPA-HF trial were enthusiastically received. Results of other ongoing trials with SGLT2i in high-risk diabetic and non-diabetic cardiovascular populations are keenly awaited and should shed more light into possible differences in clinical outcomes and prognosis, most importantly in mortality and on potential beneficial effect on MI, which remains an active topic of investigation [27, 43].

## Potential Mechanisms Behind the Cardio-renal Benefits Observed with SGLT2i

Although impressive results have been achieved with SGLT2i, there is a lack of knowledge of the mechanisms associated with the observed benefits.

SGLT2i inhibit glucose and sodium reabsorption in the kidneys, thus resulting in glycosuria. Their effects consequently include reductions in HbA1c, blood glucose levels and blood pressure (BP), but also reductions in body weight and adiposity, all mechanisms that may contribute to reducing cardiovascular risk and HF [27, 44]. The reduction in systolic and diastolic BP is reported to be about 3–7 and 2 mmHg, respectively, and seems to be independent of disease status or treatment with antihypertensive drugs [45]. Also, a reduction in body weight is consistently observed in individuals taking SGLT2i, but the magnitude of weight loss is modest (1 to 3 kg) both in T2D and in obese patients without diabetes, due to counter-regulatory mechanisms striving to maintain body weight. It is unknown whether such effects can translate into reduced cardiovascular disease events, including HF [45].

However, the evidence indicates that the cardioprotective benefits behind SLGT2i go beyond roles in glycemia, BP control, and weight loss. Firstly, the glucose and BP-lowering effects of SGLT2i compared with placebo are not sufficient to explain the outcomes observed in randomized clinical trials (RCT). Secondly, if the beneficial effects of SGLT2i were due exclusively to glycaemic or BP control, these effects should impact all CV outcomes. Although SGLT2i have a significant effect on the prevention of HF events, they are neutral in preventing atherothrombotic events such as stroke, with only a possible modest effect on MI. A subanalysis from DECLARE-TIMI 58 suggested a reduction in type 2 MI, possibly by ischemic and not anti-thrombotic mechanisms [27, 43]. Additionally, the beneficial effects of SLGT2is are seen at similar proportions across patients with different levels of HbA1c and eGFR.

**Fig. 1:**
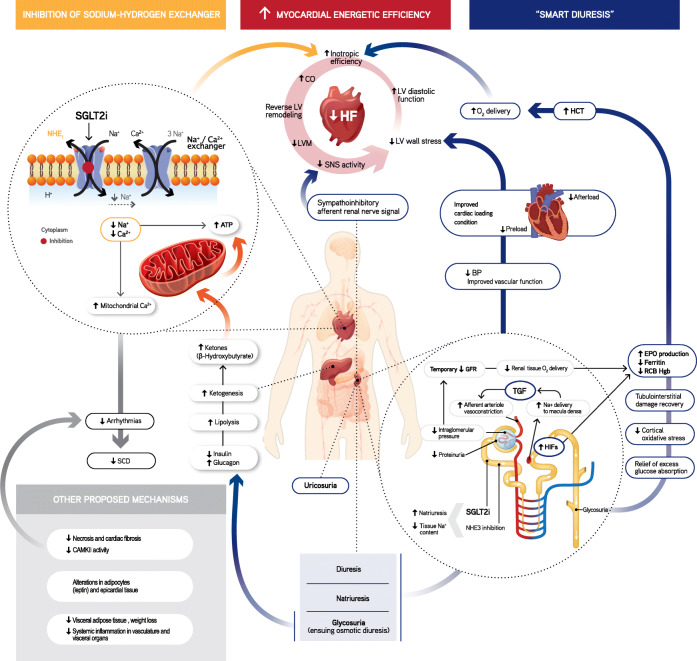
Overview of postulated cardio-renal SGLT2i translational mechanisms and observed clinical outcomes. Abbreviations: ATP, adenosine triphosphate; CO, cardiac output; GFR, glomerular filtration rate; BP, blood pressure; EPO, erythropoietin; Glu, glucose; HCT, haematocrit; MCU, mitochondrial calcium uniporter; Na^+^, sodium; Ca^2+^, calcium; NHE; sodium hydrogen exchanger; NHE1 sodium-hydrogen exchanger 1; NHE3, sodium-hydrogen exchanger 3; SCD, sudden cardiac death; SGLT2i, sodium-glucose co-transporter 2 inhibitor; SNS, sympathetic nervous system; TGF, tubuloglomerular feedback; LV, left ventricle; LVM, left ventricle mass; O_2_, oxygen; RBC Hg, red blood cell haemoglobin; HIFs, hypoxia inducible factors; SCD, sudden cardiac death; CAMKII, calcium/calmodulin-dependent protein kinase II (adapted from refs. 46; 70; 71; 73; 75; 80; Verma A, et al. J Am Coll Cardiol.. 2018; doi: 10.1016/j.jacc.2017.12.034.; Arjun S, et al. Cardiovasc Res. 2019; doi: 10.1093/cvr/cvz105.; Mazer CD, et al. Circulation. 2019; doi: 10.1161/CIRCULATIONAHA.119.044235.; Ottolia M, et al. J Mol Cell Cardiol. 2013; doi: 10.1016/j.yjmcc.2013.06.001.)

Several hypotheses have thus been postulated to explain the cardio-renal outcomes observed with SLGT2i, beyond effects on glycemia, BP and weight loss (Fig. 1).

### The “Super Fuel” Hypothesis

The healthy myocardium is metabolically “omnivorous” and able to switch between different sources of energy; it can use carbohydrates, ketones, lactate and certain amino acids as fuel, but utilizes preferentially free fatty acids (FFA) for energy production, which yield substantial amounts of energy in the form of adenosine triphosphate (ATP) molecules, albeit at the expense of higher oxygen consumption [47]. Ketone bodies may also be used by the myocardium as the most energy-efficient fuel source, producing the largest number of ATP molecules at the lowest oxygen expense.

In the diseased myocardium, there is an increased uptake of glucose and FFA into the cytosol, but this becomes uncoupled from their uptake and oxidation in the mitochondria, leading to an accumulation of metabolic intermediates, ultimately resulting in toxicity [48]. In patients treated with empagliflozin the induced glycosuria results in lower plasma glucose and insulin levels, with concomitant increased plasma glucagon (resembling a fasting state), leading to enhanced lipid mobilization [49, 50]. The lower ratio of insulin/glucagon at the portal vein and the increased circulation of FFA stimulate ketogenesis in the liver [51].

This hypothesis, suggested by previous authors as the “thrifty substrate” hypothesis, postulates that SGLT2i cause a mild but persistent increase in the production of ketone bodies, in particular beta-hydroxybutyrate, which becomes, along with FFA, the main substrates for ATP production in the myocardium, in detriment of glucose. Because ketone bodies are more energy-efficient than FFA, this shift greatly improves the energetic efficiency of the heart [52] and reduces cytotoxicity [48].

Although animal studies have been conducted to test this hypothesis, it still lacks confirmatory clinical data to support it. A study in a non-diabetic porcine model subjected to MI showed that empagliflozin increases myocardial consumption of ketones at the same time that it reduces glucose consumption, with increased myocardial energetics leading to reverse remodelling at anatomical, metabolic and neurohormonal levels [46]. The same investigators are currently conducting a trial with empagliflozin (the EMPA-TROPISM study [53]), attempting to translate these results into the clinical arena.

### The Sodium-Hydrogen Exchanger Hypothesis

One alluring hypothesis that has been put forward is that SLGT2i may offer cardio-renal benefits by directly binding to and inhibiting the sodium-hydrogen exchangers (NHE) in the heart and kidney [54]. The NHE1 isoform is ubiquitously distributed and is the predominant isoform expressed in the heart [55], whereas NHE3 expression is limited to epithelial cells of the gut and kidney, being responsible for most of the sodium reuptake after glomerular filtration [56, 57]. In HF, the activity of NHE1 in cardiomyocytes is markedly increased, leading to higher concentrations of Na^+^ in the cytosol, which in turn triggers an increase in intracellular Ca^2+^ and ultimately lead to cardiomyocyte injury and cardiomyopathy [54].

Experimental models have shown that SLGT2i directly bind to NHE1 in cardiomyocytes, reducing cytoplasmic Na^+^ and Ca^2+^ levels [58–60]. It should be noted that the SLGT2 transporter is not expressed in the heart [61]; thus, SLGT2i cannot exert their action in cardiomyocytes via SLGT2 inhibition. It has been postulated that SLGT2i may also downregulate the activity of NH3 in the proximal tubule [62]. Animal models have shown that NH3 expression is increased in HF as a result of upregulation of mineralocorticoids, leading to fluid retention and peripheral oedema [63, 64]. Mineralocorticoid antagonists such as spironolactone inhibit both NHE1 and NHE3 and ameliorate experimental models of HF [65–71]. Interestingly, in the EMPA-REG OUTCOME trial, the CV benefits with empagliflozin were attenuated in patients receiving spironolactone at baseline [29]. However, the same effect was not observed in the DAPA-HF trial, where over 70% of the population received background therapy with aldosterone antagonists [35].

### The “Smart Diuretic” Hypothesis

The “smart diuretic” hypothesis suggests that the favourable effects observed with SGLT2i are in part due to their more selective diuretic effects. SGLT2i have unique diuretic properties whereby they modulate the function of the proximal tubule, leading to natriuresis, glycosuria and ensuing osmotic diuresis [72]. The consequent sodium and volume reductions would result in lower preload and afterload, leading to improved cardiac loading conditions [73]. The proximal tubule action and natriuretic effect act as stimuli for tubuloglomerular feedback, resulting in afferent arteriolar vasoconstriction, thus lowering glomerular hypertension, and likely causing an antiproteinuric effect [73]. Besides volume contraction, SGLT2i have also been shown to increase haemoconcentration, possibly also associated to intrinsic renal mechanisms, such as the recovery of tubulointerstitial hypoxia and increased erythropoietin (EPO) production, mechanisms that require further clarification [74, 75]. It may be hypothesized that the haemoconcentration can lead to increased oxygen delivery to myocardial tissue and serve as a complementary mechanism to the “super fuel” hypothesis, further enhancing myocardial efficiency [52]. Furthermore, it has been proposed that SGLT2i have the ability to selectively reduce interstitial fluid, a property unique to this class, which may be particularly relevant for patients with congestive HF and interstitial oedema [72]. This differs from the drastic reduction in intravascular volume observed with loop diuretics, which may lead to compensatory mechanisms and neurohormonal activation, associated with deleterious effects [76]. Alternatively, a sympathoinhibitory afferent renal nerve signal is another possible mechanism to explain the absence of SGLT2i activation on the sympathetic nervous system, typically activated with diuretic therapies [77]. Other differences between SLGT2i and traditional diuretics include their uricosuric effect [78], as well as their ability to improve endothelial function and aortic stiffness [79–81].

### Other Proposed Mechanisms

Multiple other mechanisms have been proposed to explain the early cardio-renal benefits of SGLT2i, including hypotheses that rely on leptin, calcium-calmodulin inhibition, visceral adipose tissue loss and direct vascular (arterial rigidity and central pressure) effects. The effect of SGLT2i on the secretion of leptin (which contributes to the retention of sodium and to the cardiac and renal fibrosis present in patients with obesity-related HFpEF) may reduce Na^+^ retention and the accumulation of visceral adipose tissue, namely epicardial fat, and thus ameliorate the effects of systemic inflammation on the vasculature and visceral organs [82, 83]. The recently published EMPA-HEART CardioLink-6 trial [84] added information to the SGLT2i CVOTs, with the inclusion of imaging parameters evaluations. EMPA-HEART aimed to determine whether the CV benefits of SGLT2i could be secondary to a reduction in LV mass, an important and independent predictor of MI, HF and mortality. Individuals with T2D, coronary artery disease and a normal LV mass index (LVMi), representative of the EMPA-REG OUTCOME cohort, were included. After six months, there was a significant reduction in LVMi (measured by cardiac magnetic resonance imaging) associated with empagliflozin [84]. Importantly, the observed reduction in LVMi appeared to occur without reductions in LV volumes, thus reflecting an overall reduction in LV wall thickness, with a greater magnitude of regression observed in patients with higher LVMi at baseline. However, the mechanisms behind the reduction in wall thickness remain to be elucidated, possibly relating to changes in interstitial water content or reduced cardiomyocyte mass. Other evidence from small studies (Moura, B et al. Empagliflozin: effects on the heart and vessels. P2059. ESC Congress 2019. 31 August—04 September 2019, Paris, France) showed that SGLT2i induces a decrease in atrial volume and an improvement in diastolic function beyond reverse remodelling [85].

Key translational mechanisms on CV physiology which are currently in need for further data with SGLT2i are listed in Table 4.

**Table 4:** Key translational mechanisms in need for further data with SGLT2i

▪ Serum cardiac and renal biomarkers (e.g. NT-proBNP*, Gal-3, soluble ST2, other)	
▪ Electrophysiological mechanisms	
▪ Effects on sympathetic nervous system	
▪ Effects on neurohormonal responses	
▪ Effects of uric acid reduction	

*iSGLT2 effects on NT-proBNP levels have been contradictory and require further confirmation in large RCTs of HF populations

*Abbreviations*: *NT-proBNP*, N-terminal pro–B-type natriuretic peptide; *Gal-3*, galectin-3; *ST2*, suppression of tumorigenicity

In conclusion, the various described hypotheses and mechanisms are not mutually exclusive and may all play a part in the observed cardio-renal outcomes with SGLT2i. Most likely, the hemodynamic, metabolic and tissue/cellular mechanisms work synergistically in promoting the observed benefits.

The ongoing mechanistic trials being conducted mostly in HF populations should be able to shed more light on the SGLT2i effects in myocardial bioenergetics, biomarkers and remodelling parameters (Table 5) [53, 86–89]. In addition, the ongoing trials evaluating functional capacity and quality of life (QoL), including both HFrEF and HFpEF patients (Table 3) [39, 40] should bring further insights to guide clinical practice.

**Table 5:** Summary of ongoing or completed mechanistic trials of SGLT2i

	EMPA-VISION	EMPA-TROPISM [53]	EMBRACE-HF	ELSI	Empire HF [86]	EMPA	ERA-HF	RECEDE-CHF [87]	SUGAR	EMMY	REFORM [88]	DAPACARD [89]	DAPA-Shuttle1	EMMED-HF	ERADICATE-HF	ERTU-GLS
NCT number	03332212	03485222	03030222	03128528	03198585	03027960	03271879	03226457	03485092	03087773	02397421	03387683	04080518	04071626	03416270	03717194
Active substance/comparator	Empa/Placebo	Empa/Placebo	Empa/Placebo	Empa/Placebo	Empa/Placebo	Empa/Placebo	Empa/Placebo	Empa + furosemide/Placebo + Furosemide	Empa/Placebo	Empa/Placebo	Dapa/Placebo	Dapa/Placebo	Dapa/Placebo	Ertu/Placebo	Ertu/Placebo	Ertu/Placebo
Population	HFrEF or HFpEF with or without T2D	HFrEF with or without T2D	HFrEF or HFpEF with or without T2D	HFrEF or HFmrEF with or without T2D	HFrEF with or without T2D	HFrEF or HFpEF with T2D	HFrEF with T2D	HFrEF with T2D	HFrEF with T2D	Acute MI with or without T2D	HFrEF or HFpEF with T2D	HpEF with T2D	HFpEF or HFrEF with T2D	HFpEF with T2D	HFpEF or HFrEF with T2D	HFpEF or HFrEF with T2D
Aim	Effects of Empa treatment on cardiac physiology and metabolism (energetics) in HF patients	Efficacy and safety of Empa in non-diabetic HF patients	Effects of Empa on hemodynamic parameters (pulmonary artery pressures) in patients with HF	Effect of Empa on reduction of tissue sodium content	Effect of Empa on cardiac biomarkers, cardiac function (at rest and during stress), cardiac hemodynamic, renal function, metabolism, daily activity level and health-related QoL	Acute/short term effect and cardio-renal mechanisms of SGLT2 inhibition in patients with HF	Effect of Empa on the rate of arrhythmic events in HF patients	Effect of Empa on urinary volume	Effects of Empa on clinical measures of cardiac structure and function, and renal blood flow	Effect of empagliflozin after acute MI on HF	Effect of SGLT2 inhibition on LV remodelling in patients with HF and T2D	Effects of Dapa on cardiac substrate uptake, myocardial efficiency and myocardial contractile work in T2D patients	Effects of Dapa on renal concentration mechanism and mobilization of Na^+^ and fat stores	Effect of Ertu on cardiac metabolism in T2D HFpEF patients	Mechanisms whereby Ertu modifies cardio-renal interactions that regulate fluid volume and neurohormonal activation in patients with HF and T2D	Effect of Ertu on cardiac function in patients with T2D and HF
Sample size	86	80	60	84	189	50	128	23	130	476	56	53	40	52	36	120
Key inclusion criteria	– HFrEF or HFpEF, with or without diabetes – Aged 18 years or older	– Stable HF for > 3 months – LVEF < 50% – NYHA II to IV	– HFrEF or HFpEF – NYHA II to IV – Previously implanted CardioMEMS pulmonary artery pressure monitor – PA diastolic pressure ≥ 12 mmHg	– Ejection fraction < 40% or 40–49% and NT-pro BNP > 125 pg/mL and at least one structural abnormality of left atrium or ventricle	– LVEF ≤ 0.40 – eGFR > 30 mL/min/1.73 m^2^ – BMI < 45 kg/m^2^ – NYHA class I–III – If T2D - HbA1C 6.5–10%	– Stable HF – T2D – eGFR ≥ 45 mL/min/1.73 m^2^ – Chronic daily oral loop diuretic dose ≥ 20 mg furosemide equivalents	– HFrEF – NYHA ≥II – Implanted ICD, CRTD or CRTP devices – T2D – HbA1c ≥ 7% and ≤ 12%	– NYHA Functional class II–III HF with prior echocardiographic evidence of LVSD – T2D – eGFR ≥ 45 mL/min	– T2D – NYHA class II–IV – HFrEF (LVEF ≤ 40%)	– MI (in the last 72 h) – eGFR > 45 mL/min/1.73m^2^ – BP > 110/70 mmHg	– T2D – NYHA class I–III – HF with LVD – Stable HF for > 3 months	– T2D – HbA1c 6–9% – LVEF ≥ 50% – BMI ≥ 25 kg/m^2^ – eGFR ≥ 45 mL/min/1.73 m^2^	– T2D – NYHA class I or II	– Age > 18 < 75 years old – BMI > 29 < 40 4 – T2DM 6 – Stable HFpEF	– T2D – eGFR ≥ 30 mL/min/1.73 m^2^; – HbA1c 6.5–10.5%; – BMI 18.5–45.0 kg/m^2^; – BP ≤ 160/110 and ≥ 90/60 at screening – HF – NYHA II–III	– T2D – eGFR ≥ 45 mL/min/1.73 m^2^ – Stage B HF
Primary endpoint	PCr/ATP ratio	LVEDV LVESV, LVEF, LV mass CPET, 6MWT and QoL questionnaires	Change in pulmonary artery diastolic pressure	Skin sodium content	Change of plasma concentrations of NT-proBNP	Urine sodium concentrations via ion selective electrodes	Burden of premature ventricular complexes, defined as the PVCs percentage of all beats in a pre-specified period captured on ICD or CRTD/P device	Change from urinary volume	LVESVI and LV global longitudinal strain	Change of NT-proBNP levels	Changes in LVESV and LVEDV	Change in global longitudinal strain of the LV	Changes in urinary osmolyte concentration	Peak VO_2_, ml/kg/min, measured by metabolic gas exchange	Difference in proximal sodium reabsorption	Global longitudinal strain
Results/status	Estimated completion April 2020	Estimated completion December 2020	Estimated completion December 2019	Estimated completion December 2019	Estimated completion October 2019	Completed August 2017 No results available	Estimated completion June 2020	Completed January 2019 No results available	Estimated completion February 2020	Estimated completion November 2019	Completed August 2017 No results available	Estimated completion March 2019 No results available	Estimated completion April 2020	Estimated completion June 2021	Estimated completion March 2021	Estimated completion October 2020

*Abbreviations*: *6MWT*, six-minute walking test; *BMI*, body mass index; *BP*, blood pressure; *CPET*, cardiopulmonary exercise test; *CRTD*, cardiac resynchronization therapy defibrillator; *CRTP*, cardiac resynchronization therapy pacemaker; *Dapa*, dapagliflozin; *eGFR*, estimated glomerular filtration rate; *Empa*, empagliflozin; *Ertu*, ertugliflozin; *HbA1c*, haemoglobin A1c (glycated haemoglobin); *HF*, heart failure; *HFmrEF*, heart failure with mid-range ejection fraction; *HFpEF*, heart failure with preserved ejection fraction; *HFrEF*, heart failure with reduced ejection fraction; *ICD*, implantable cardioverter defibrillator; *LV*, left ventricle; *LVEDV*, left ventricular end diastolic volume; *LVEF*, left ventricular ejection fraction; *LVESV*, left ventricle end systolic volume; *LVESVI*, left ventricle end systolic volume index; *LVSD*, left ventricular systolic dysfunction; *MI*, myocardial infarction; *NT-proBNP*, N-terminal pro–B-type natriuretic peptide; *NYHA*, New York Heart Association classification; *PA*, pulmonary artery; *PCr/ATP*, phosphocreatine-to-adenosine triphosphate ratio; *PVCs*, premature ventricular contractions; *QoL*, quality of life; *SGLT2*, sodium-glucose co-transporter 2 inhibitors; *T2D*, type 2 diabetes mellitus

## Practical Considerations for SGLT2i Management in T2D Patients with HF

### SGLT2i Safety Profile

SGLT2i generally have a favourable efficacy and safety profile. The recommended doses for the different SGLT2i are reviewed in Supplementary table 1.

Genital mycotic infections are the most frequent AE reported, as well as an increase in urinary tract infections, although the later with no statistically significant differences compared with placebo. Other safety issues such as bone fractures and peripheral amputations have only been observed in one clinical trial with canagliflozin [21]. A number of rare AEs have been reported, including ketoacidosis and Fournier’s gangrene, which have led to specific warnings by regulatory agencies. Nevertheless, there are specific measures that can be previously assured to prevent and to manage these events [90] (Supplementary table 2).

### Special Considerations for Management of HF Therapies and SGLT2i

According to the 2016 ESC HF Guidelines, empagliflozin should be considered in T2D patients to delay or prevent the onset of HF and prolong life, as a class IIA, level B recommendation [1]. In the recent expert consensus update from the Heart Failure Association (HFA) of the ESC [91], it is recommended that dapagliflozin and canagliflozin should also be considered in T2D patients with established CVD or high CV risk to prevent or delay the onset of HF and HHF. These documents, however, do not specifically recommended dapagliflozin and canagliflozin to prolong life, in alignment with the recent ESC/EASD Guidelines for diabetes and pre-diabetes, where prognostic recommendations are reserved to empagliflozin based on CVOT data. Although in the HFA expert consensus, no specific recommendations for the use of SGLT2i could be made for patients with established HF, some practical considerations were highlighted. Upon initiation of an SGLT2i, an initial “dip” in eGFR can be noted in some patients (an average decrease of 3–5 mL/min), suggested as a positive marker for long-term preservation of renal function [91, 92]. This remains to be confirmed specifically in HF patients with or without T2D.

The observed effects of SGLT2i in eGFR may be similar to the effect noted upon initiation of angiotensin-converting enzyme inhibitors (ACEi) or angiotensin receptor blockers (ARB) therapy, and therefore caution may be recommended upon concomitant initiation of SGLT2i and renin-angiotensin-aldosterone system (RAAS) modulating therapies. The long-term renal preservation with both classes, however, may be synergistic considering their complementary mechanisms. Similarly, as most HF patients are managed with loop diuretics for congestion, adjustments to baseline diuretic therapy may be necessary upon SGLT2i initiation, based on adequate volume assessment and definition of volume status. Temporary discontinuation of SGLT2i and/or diuretic agents may be required to manage clinical hypovolemia and ketoacidosis (Supplementary table 2) [91]. Regarding the management of diuretics in HF, the HFA has recently published a position statement [93]. SGLT2i are described as “other potential agents” and recommended as third-line therapy in acute congestive HF for the management of diuretic resistance [93]. Importantly, no specific recommendations are considered for the concomitant use of SGLT2i and other diuretics in the ambulatory chronic HF setting, where SGLT2i therapy will most often be implemented [94]. It is worthwhile noting that the electrolyte disturbances and renal deterioration associated with traditional diuretic agents used in HF management are not observed with SGLT2i, which would facilitate clinical management.

Another challenge in the management of HF patients is the treatment-associated or underlying progression of disease-associated hypotension. As most disease-modifying HF therapies reduce BP, the addition of SGLT2i therapy (average BP reduction of 3–5 mmHg) should be implemented with caution, particularly in patients with lower baseline BP. The strategy of pre-emptively reducing doses of non-disease-modifying therapies, such as loop diuretics, may allow for a safer introduction of SGLT2i without additive hypotension or dehydration, as is currently recommended for achieving target doses of disease-modifying agents. As previously highlighted, most patients in the DAPA-HF trial were well treated with background HF therapies (including over 93% on diuretics) and no mandatory adjustments to baseline therapy were required by protocol; unexpectedly, the addition of dapagliflozin demonstrated tolerability similar to placebo.

### Considerations for Management of Antidiabetic Agents in Patients with HF

According to the 2016 ESC HF Guidelines, glycaemic control should be implemented in a gradual and moderate manner, and metformin is recommended as the first-line oral hypoglycaemic drug for HF patients [1]. Considering this document, as well as more recent evidence and recommendations from the American Heart Association scientific statement on T2D and HF [95], in addition to the 2019 ESC/EASD diabetes guidelines [32], Table 6 summarizes the circumstances to consider when selecting and using antidiabetic agents in patients with HF.

**Table 6:** Considerations for the management of glucose-lowering medications in patients with HF (adapted from refs. 1, 32, 90)

Antidiabetic agent	Considerations for management of diabetes in HF patients
Metformin	Safe to use at all stages of HF in patients with preserved or moderately reduced renal function (GFR > 30 mL/min). Lower risk of death and HHF compared with sulphonylureas and insulin. Should be discontinued in patients presenting acute conditions associated with lactic acidosis (e.g. cardiogenic or distributive shock).
Sulphonylurea	Limited data concerning the development of HF in individuals with DM. Effects on HF outcomes have been inconsistent. Should be used with caution.
Insulin	Associated to weight gain and risk of hypoglycaemia. May exacerbate fluid retention, leading to HF worsening. Should be used with caution; close monitoring.
Thiazolidinediones (glitazones)	Cause sodium and water retention. Increased risk of worsening HF and rates of HF hospitalization in individuals with DM without HF. Not recommended in patients with symptomatic HF, or at high risk for developing HF.
Long-acting glucagon-like peptide 1 receptor agonists (GLP-1)	Low risk of hypoglycaemia. Safe to use and improve glycaemic indices, but not beneficial in preventing HF in patients at risk. Neutral effect on HHF. Use may be considered.
Dipeptidylpeptidase-4 inhibitors (DPP4is; gliptins)	Improved glycaemic indices but no evidence on cardiovascular benefit. Can increase the risk of HHF in patients with DM at high cardiovascular risk (i.e. saxagliptin, possible with alogliptin). Increases in LV volumes were observed with vildagliptin. Neutral effects for sitagliptin and linagliptin. Should not be considered in patients with HF, or at high risk for developing HF.
Sodium-glucose co-transporter type 2 inhibitors (SGLT2i)	First class of glucose-lowering agents to demonstrate HF hospitalization risk reduction in patients with DM. Recommended for patients with T2D to reduce HF risk (class IA recommendation). Promising for treatment of established HF in patients with and without DM.

*Abbreviations*: *DM*, diabetes mellitus; *GFR*, glomerular filtration rate; *HF*, heart failure; *HHF*, hospitalization for heart failure; *LV*, left ventricular; *T2D*, type 2 diabetes mellitus

## Unmet Medical Needs and Conclusions

Despite the close pathophysiological relation between T2D and HF, cardiovascular outcome trials with SGLT2i were not designed to test their efficacy and safety, specifically in HF patients. Only a small proportion of patients in the EMPA-REG OUTCOME trial, CANVAS program and DECLARE-TIMI 58 trial had a diagnosis of HF at baseline (and in those who had, the HF phenotype was not initially characterized). DAPA-HF was the first in class RCT to demonstrate a significant impact of an SGLT2i vs. placebo, on top of optimal medical therapy, in terms of morbidity and mortality in patients with HFrEF, irrespective of the presence of T2D [35, 38].

Similarly, the smaller DEFINE-HF trial supported these results by showing beneficial effects of dapagliflozin on HF-related QoL, and symptoms in HFrEF patients [40], and dapagliflozin has now been given a “fast track” status by the FDA for a proposed indication to reduce CV death or worsening HF.

The results from ongoing SGLT2i HF-dedicated outcomes trials are expected to lead to a growing potential for SGLT2i use in HF clinical practice (in the same way that the EMPA-REG OUTCOME trial drove a major shift in T2D management) and will answer the remaining questions of whether the results observed in DAPA-HF extend to other SGLT2i, and if the beneficial effects also include the HFpEF population.

Additional potential usage of SGLT2i may be anticipated for the treatment of acute HF (AHF), which is the major cause of HHF, and for which there are no currently available therapies that improve clinical outcomes [96]. The EMPA Acute Heart Failure, EMPAG-HF and EMPULSE trials are underway to study the effects of SGLT2i on clinical outcomes in the AHF population (ClinicalTrials.gov Identifiers: NCT03554200, NCT04049045 and NCT04157751, respectively), while the EMPA-RESPONSE study has been published recently [97].

Another limitation of the RCT evidence with SGLT2i is that the reported trials were designed to test CV outcomes, but not SGLT2i CV actions, for which mechanistic clinical trials are currently underway (Table 5). The results of ongoing trials are eagerly awaited by the scientific HF community, especially considering the growing number of HF patients worldwide.

In summary, the evidence to date tells us that SGLT2i offer cardioprotection for HF patients (particularly those with HFrEF) and also to those at risk for developing HF, benefits that appear to be independent of glycaemic control, and that are observed in populations with and without T2D. The mechanisms underlying this protection can act in various ways and be complementary or synergistic.

Deepening this knowledge may help to identify more targeted therapies in the future, according to the patients’ overall CV and HF profile.

Undoubtedly, a new chapter of this history has begun.

## Electronic Supplementary Material

ESM 1 (DOCX 27 kb)
