# Supplementary material for: Sodium-Glucose Co-transporter 2 Inhibitors in the Failing Heart: a Growing Potential
Source: Cardiovasc Drugs Ther. 2020 Apr 30;34(3):419–36. doi: 10.1007/s10557-020-06973-3 (PMC7242490; doi:10.1007/s10557-020-06973-3)
Supplement: Supplementary file 1 — (DOCX 27 kb) [file 10557_2020_6973_MOESM1_ESM.docx]

**Supplementary material**

**Suppl. Table 1.** SGLT2i recommended doses and action on specific outcomes

| **Drug** | **Initial dose** | **Patients requiring additional glycaemic control** | **eGFR values for initiation and discontinuation** | **Effects on glycaemic control** | **Effects on body weight** | **Effects on blood pressure** |
| --- | --- | --- | --- | --- | --- | --- |
| **Canagliflozin** | 100 mg PO once daily taken before the first meal of the day.  If used as add-on, lower doses of insulin or sulphonylurea may be considered to reduce the risk of hypoglycaemia. | Up to 300 mg PO per day if the initial dose is well tolerated and eGFR ≥60 mL/min/1.73 m² | Start: eGFR ≥60 mL/min/1.73 m²  Stop: eGFR <45 mL/min/1.73 m² | Reduction in HbA1c seen in placebo-controlled studies as monotherapy and in different combinations with metformin, sulphonylurea, pioglitazone, insulin and sitagliptin.  Difference from placebo as monotherapy (%):  100 mg: **-0.91** (-1.09; -0.73 95% CI)  300 mg: **-1.16** (-1.34; -0.98 95% CI) | Reduction in body weight seen in placebo-controlled studies as monotherapy and in different combinations with metformin, sulphonylurea, pioglitazone, insulin and sitagliptin.  Reduction in body weight seen in active-controlled studies in comparison with glimeripide and sitagliptin.  Difference from placebo as monotherapy (kg):  100 mg: **-2.2**  (-2.9; -1.6 95% CI)  300 mg: **-3.3**  (-2.7; -1.6 95% CI) | Reduction in systolic BP seen in placebo-controlled studies as monotherapy and in different combinations with metformin, sulphonylurea, pioglitazone, insulin and sitagliptin.  Reduction in systolic BP seen in active-controlled studies in comparison with glimeripide and sitagliptin. |
| **Dapagliflozin** | 5 mg PO once daily at any time of day, with or without food  If used as add-on, lower doses of insulin or sulphonylurea may be considered to reduce the risk of hypoglycaemia. | Up to 10 mg PO per day  No dose adjustment is required based on renal function | Start: eGFR ≥60 mL/min/1.73 m²  Stop: eGFR <45 mL/min/1.73 m² | Reduction in HbA1c seen as monotherapy and in different combinations with metformin, glimeripide, sulphonylurea, sitagliptin and insulin.  Difference from placebo as monotherapy (%):  10 mg: **-0.66**  (-0.96; -0.36 95% CI) | Reduction in body weight seen as monotherapy and in different combinations with metformin, glimeripide, sulphonylurea, sitagliptin and insulin.  Difference from placebo as monotherapy (kg):  10 mg: **-0.97**  (-2.20, -0.25 95% CI) | Reduction in systolic and diastolic BP seen in placebo-controlled studies.  Reduction in systolic BP in hypertensive T2D patients |
| **Empagliflozin** | 10 mg PO once daily, with or without food  If used as add-on, lower doses of insulin or sulphonylurea may be considered to reduce the risk of hypoglycaemia. | Up to 25 mg PO per day if the initial dose is well tolerated and eGFR ≥60 mL/min/1.73 m² | Start: eGFR ≥60 mL/min/1.73 m²  Stop: eGFR <45 mL/min/1.73 m² | Reduction in HbA1c seen in monotherapy and as dual therapy with metformin, pioglitazone, sulphonylurea, DPP-4 inhibitors and insulin.  Difference from placebo as monotherapy (%):  10 mg: **-0.74**  (-0.90; -0.57 97.5% CI)  25 mg: **-0.89**  (-1.01; -0.69 97.5% CI) | Reduction in body weight seen in monotherapy and as dual therapy with metformin, pioglitazone, sulphonylurea, DPP-4 inhibitors and insulin.  Difference from placebo as monotherapy (kg):  10 mg: **-1.93**  (-2.48; -1.38 97.5% CI)  25 mg: **-2.15**  (-2.70; -1.60 97.5% CI) | Reduction in systolic and diastolic BP seen in monotherapy and as dual therapy with metformin, pioglitazone, sulphonylurea, DPP-4 inhibitors and insulin |
| **Ertugliflozin** | 5 mg PO once daily  When used in combination with insulin or an insulin secretagogue, a lower dose of insulin or the insulin secretagogue may be required to reduce the risk of hypoglycaemia | Up to 15mg PO once daily | Start: eGFR ≥60 mL/min/1.73 m²  Stop: eGFR <45 mL/min/1.73 m² | Reduction in HbA1c seen as monotherapy and in different combinations with metformin and sitagliptin.  Difference from placebo as monotherapy (%):  5 mg: **-1.00** (-1.20; -0.80 95% CI)  15 mg: **-1.20** (-1.40; -0.90 95% CI) | Reduction in body weight seen as monotherapy and in different combinations with metformin and sitagliptin.  Difference from placebo as monotherapy (%):  5 mg: **-1.80** (-2.60; -0.90 95% CI)  15 mg: **-2.20** (-3.00; -1.30 95% CI) | Reduction in systolic BP seen in placebo-controlled studies as monotherapy |

**Abbreviations: BP**, blood pressure; **CI**, confidence interval; **DPP-4**, dipeptidyl peptidase 4; **eGFR**, estimated glomerular filtration rate; **HbA1c**, haemoglobin A1c (glycated haemoglobin); **SGLT2i**, Sodium-glucose co-transporter 2 inhibitors; **T2D**, type 2 diabetes mellitus.

**Suppl. Table 2.** SGLT2i safety issues and management tips

| **Adverse Event** | **Frequency** | **Additional information** | **Tips for patients** | **Tips to manage** |
| --- | --- | --- | --- | --- |
| **Genital mycotic infections**  **(vaginitis, balanitis)** | Common | Mild to moderate intensity, reported to occur in the first 24 weeks of treatment; most common in women and circumcised men | Pre-emptively inform patients and reinforce adequate patient genital/ perineal hygiene; may experience polyuria (although no increased risk of urinary tract infections vs placebo) | Respond well to topical antifungal treatment (occasionally oral flucanozole may be needed) and rarely lead to SGLT2i discontinuation |
| **Volume depletion and hypotension** | Rare, except in elderly patients | Closely assess elderly patients prior to therapy initiation | Inform patients about signs of orthostatic hypotension and dizziness; monitor BP and HR daily | Assess volume status and BP prior to SGLT2i initiation and correct hypovolemia, if needed (reduce loop or thiazide diuretic doses, as deemed appropriate); assess need of any non-disease modifying hypotensive therapies |
| **Acute kidney injury** | Rare, except if underlying volume depletion | May predispose patients to experience a temporary initial reduction in GFR, in particular those on concomitant diuretics and/or RAAS inhibitors; SGLT2i associated with long-term renal preservation | Inform patients to monitor any sudden reduction in urine output | Adequate assessment of volume status and correction of hypovolemia prior to SGLT2i initiation; usually, transitory reduction in GFR during therapy initiation; close monitoring of renal function is recommended in HF patients |
| **Hypoglycaemia** | May be common in patients receiving background therapy with sulphonylureas or insulin; low risk with SGLTi therapy alone | Pre-emptive significant reduction in insulin dose is not recommended | Inform patients on recognition and management of hypoglycemia | Consider decrease in insulin dose by 10% upon SGLT2i initiation |
| **Ketoacidosis** | Rare | The risk increases about two-fold during the first 180 days after initiating an SGLT2i; may occur at lower than expected glucose levels (euglycemic DKA) | Discourage excessive alcohol intake and low carbohydrate diets  Pre-emptively inform patients about potential symptoms: fruity odour on breath, thirst, polyuria, nausea, vomiting, abdominal pain, confusion, and fever | Temporarily or  permanently discontinue treatment with SGLT2i and/or diuretic agents; Hold dose if acutely ill, limited oral intake or 3 days before surgery |
| **Bone fractures and amputations** | Rare | Canagliflozin was associated with an approximately 2-fold increased risk of  lower limb amputation (mainly toes and metatarsal)  Increased risk among patients with previous amputations or in patients with peripheral artery disease | Pre-emptively inform patients to perform regular foot exams and to see a podiatrist at least annually | Regular foot exams  Avoid treatment with SGLT2i in patients with previous amputation or active foot ulceration |
| **Fournier’s gangrene** | Very rare | Serious and potentially disfiguring infection | Pre-emptively inform patients and reinforce reporting if they develop a combination of symptoms of pain, tenderness, redness, or swelling of the genitals or the area between the genitals and the anus with fever or feeling of general malaise | If suspected, start treatment with broad-spectrum antibiotics and surgical debridement if necessary.  Stop treatment with SGLT2i |

**Abbreviations: DKA**, diabetic ketoacidosis; **SGLT2i**, Sodium-glucose co-transporter 2 inhibitors.
